# Enhancement, ethics and society: towards an empirical research agenda for the medical humanities and social sciences

**DOI:** 10.1136/medhum-2015-010718

**Published:** 2015-08-10

**Authors:** Martyn Pickersgill, Linda Hogle

**Affiliations:** 1 Usher Institute for Population Health Sciences and Informatics, Edinburgh Medical School, University of Edinburgh, Edinburgh, UK; 2 Department of Medical History and Bioethics, University of Wisconsin-Madison, Madison, Wisconsin, USA

**Keywords:** Public health, Health policy

## Abstract

For some time now, bioethicists have paid close attention to issues associated with ‘enhancement’; specifically, the appropriate use and regulation of substances and artefacts understood by some to improve the functioning of human bodies beyond that associated with ‘normal’ function. Medical humanities scholars (aside from philosophers and lawyers) and social scientists have not been frequent participants in debates around enhancement, but could shine a bright light on the range of dilemmas and opportunities techniques of enhancement are purported to introduce. In this paper, we argue that empirical research into the notion and practice of enhancement is necessary and timely. Such work could fruitfully engage with—and further develop—existing conceptual repertoires within the medical humanities and social sciences in ways that would afford benefit to scholars in those disciplines. We maintain that empirical engagements could also provide important resources to bioethicists seeking to regulate new enhancements in ways that are sensitive to societal context and cultural difference. To this end, we outline an empirical agenda for the medical humanities and social sciences around enhancement, emphasising especially how science and technology studies could bring benefits to—and be benefitted by—research in this area. We also use the example of (pharmaceutical) cognitive enhancement to show how empirical studies of actual and likely enhancement practices can nuance resonant bioethical debates.

## Introduction

Biomedical and technological developments, regarded as having the capacity to improve the body's appearance or function, are often termed ‘enhancements’.^1–3^^i^ Over the last 15 years or more, bioethical and policy discourses have questioned the appropriateness of enhancements for individuals and societies.^4^ ^5^ Discussions have turned on the problems of differentiating enhancement from therapy, the hubris of changing nature and the potential for exacerbating social inequalities. Such questioning has resulted in sometimes acrimonious debate playing out in journals, conferences and in the meeting rooms of learned societies and policy advisory committees—as well as in more public arenas. These widely circulating questions and concerns around enhancement are, no doubt, important matters for consideration, but we believe they are not the only issues of import. More needs to be understood about the social, political and historical contexts in which ‘enhancements’ arise, as well as the reception and uptake of specific drugs or devices.^6–8^ Further, the potential of new technologies to reconfigure health, medicine, social relations and subjectivities is also relevant to bioethical debate. Yet, if and when this complex issue appears within bioethics discourse, it is often theorised in isolation from other traditions that have taken the relationships between materials, bodies and societies to be key foci.

In this article, we suggest that the empirical medical humanities and social sciences can contribute meaningfully to societal discourse around enhancement in two key ways. First, and most importantly, by attending to issues less commonly considered in current debates. Second, by providing methodologically robust studies of actual practices involving technologies and chemicals regarded as enhancing (which might intersect directly with—and in some cases challenge—bioethical deliberation).^9^ We take these matters in turn, with the first and substantive focus of this paper examining how research foci within the medical humanities and social sciences might (re)direct analytical attention to the tools, bodies and institutions that populate debates around enhancement. Then, we move to a more specific account of ‘cognitive enhancement’ using pharmaceuticals meant for attention disorders. These are employed as a case study of empirical engagement with a matter of bioethical concern, in order to consider the benefits to bioethics that such analyses might afford. Debates around drugs taken to have enhancing properties to the human brain are wide-ranging; such pharmaceuticals attract broad attention due to the wider social and legal contexts of their use and the potential implications for personal responsibility and liability that come with this. An exploration of extant studies is indicative of new empirical research trajectories that might be considered, and the salience of such scholarship for bioethical deliberation.

## Exploring new terrain

We outline some research questions and directions that might contribute to research on enhancement that intersects with existing bioethics scholarship. As a consequence of our own inclination towards the field of science and technology studies (STS), we start with specifying questions that direct the gaze of scholars to the objects around which bioethical debate circulates (eg, pharmaceuticals), before considering the sociotechnical networks that enable, legitimise and sustain their production and consumption.

Like others, we feel that the substantial body of interdisciplinary STS scholarship on the social shaping of technology is of immediate relevance and import to our considerations.^2^ ^6^ ^8^ ^10^ Such work seeks to open up the ‘black box’ of technology and consider the ways in which a range of social and economic factors construct the eventual form and feel of an object. In the case of enhancement, we might ask: what ideas about society, of the body, and of individual hopes and fears are imagined and/or channelled within processes of innovation? What values—clinical, scientific, economic, societal and so on—are ‘inscribed’ in particular substances and technologies deemed to be enhancing?^11^ How are the practices of potential users configured in the process?^12^ Scrutiny of the circulation and transformation of different kinds of value and values within the innovation processes producing technological enhancements will likely represent an interesting case for scholars who have attended to these issues in more straightforwardly biomedical contexts (such as in drug trials, trial recruitment and marketing).^13^ Disaggregating the different values and practices of valuation constitutive of the innovation and consumption of materials purchased (in formal, grey or black markets) for their enhancing properties also serves to trouble the more essentialist claims of some bioethical commentators who present these as unproblematic social and individual goods.^3^ ^6^ ^8^ ^10^ ^14^

### Regulating biomedical lives

As a range of scholars have shown, economic value relates closely to how any new tool, technology or drug is apprehended by law, constituted through legal processes and regulated on the market.^15^ Questions of law and regulation, then, are central to innovation processes, both during development and after the finished products are released. Recent work at the interface of regulatory studies and STS has illustrated how particular modes of governance are part of the societal context that can be built into technologies, helping to shape their design and mode(s) of delivery.^16–18^ Sheila Jasanoff, for example, uses cross-cultural case study methods to demonstrate how political cultures affect the way societies assess evidence, evaluate risk and engage with publics around innovations in science and technology.^19^ Such perspectives could contribute considerably to understanding the differences regarding how policy issues around enhancements are framed, as well as what—and whose—values become embedded in the institutions governing their use.

Social histories of technologies are also often omitted from studies of ‘enhancements’, but can be key to understanding the interpretation of certain technologies. For example, the burgeoning science of optogenetics uses optics and gene modification techniques to control the activity of specific cells.^ii^ In addition to the promise of modulating cell function in neurological disorders, scientists suggest they can alter addictions, depression and mood disorders and other behavioural and psychosocial conditions. Previous controversies over gene therapy and psychosurgery will surely colour how such emerging techniques will be viewed from both regulatory and ethical perspectives.^20^ ^21^ Additionally, popular culture, including science fiction themes of mind control, have been known to affect legal frameworks in which such brain interventions would be viewed for therapeutic purposes, much less enhanced function. There has already been considerable scholarly interest in the interactions of law and neuroscience, particularly in light of questions of altered autonomy, identity, personal responsibility and liability with the use of brain stimulation techniques.^22–24^ Accordingly, it will be fruitful to examine how legal processes shape the production and deployment of tools and substances that are understood to enhance human bodies and cognition. This is in terms of how *actual* regulation directs innovation, and if and how imaginaries of *future* governance inform the development and marketing of objects framed as enhancements. Court cases where these figure would likewise be important to examine, in terms of how object ontologies (eg, purported nature of particular drugs and devices, and hence, the ‘appropriate’ uses to which they are put) and subjective properties (eg, authenticity, personhood, etc.) are co-produced. It is also important to attend to the ways that such innovations are situated within so-called knowledge-intensive societies, with their emphasis on performance, productivity, competition and connectivity. Markets are likely to help shape legal and regulatory environments as much as the technologies themselves.

Documenting how ‘enhancements’ are dealt with in clinical practice especially—including who is empowered to control access to them, and why—will be a key empirical task for those in the medical humanities and social sciences. Not enough is known about how physicians represent to patients (and themselves consider) the benefits, risks and harms of potentially enhancing products and treatments, nor how ‘legitimate’ use is configured.^25^ Further to reflecting on the philosophical or policy implications of how products might be classified as therapeutic or enhancing, scholars might ask: what comes to count in how health professionals (not solely medical practitioners) decide whether the use of one particular drug or technology counts as ‘therapy’ or as ‘enhancement’? For instance, do physicians consider the moral or legal implications of prescribing attention deficit-hyperactivity disorder (ADHD) drugs for off-label purposes, including enhancing cognition? To what extent are they aware of how patients may be using such prescribed drugs, including selling them to others?^26^ The literature on ethical decision-making in clinical practice will afford benefit to empirical analysis of such questions, which will in turn enrich scholarship around ethics-in-practice.^27^ ^28^

Ethnographic and conversation analytic work might usefully examine the physician–patient dance involved in representing a subjective state as a legitimate target of optimisation, not least to cast further light on the cultural logics shaping discourses of legitimacy today.^29^ In cases where physicians may be reluctant to prescribe ‘enhancements’ to individuals who ask for them, where do individuals who wish to consume these then turn? Research exploring such issues may have much to offer sociolegal studies in terms of understanding how grey markets are formed and negotiated by a range of individuals. Further, interviews and other methodological strategies might be employed to examine how users construct the benefits, risks and harms of substances and tools used to enhance human bodies (in particular, where access is achieved beyond the clinic, such as via the internet). Users may employ these notions in ways that are quite distinct from those featuring within biomedical and bioethical discourses, and the regulatory regimes that are in part constituted through these (as we will show below—drawing on a range of work—for the case of cognitive enhancement).^8^ For example, comparative work between prescription and non-prescription ‘enhancements’ (such as the use of nicotine replacement patches as stimulants) regarding publics’ constructions of risk would be both timely and have import for scholarly interrogations of the production of risk, harm and safety in other (non-)medical domains. Since public engagement plays an important role in in shaping policy approaches to new and emerging technologies, consideration will be required of how this is leveraged as a regulatory device to either promote or curtail the use of supposed enhancements.^30^

Relatedly, more critical work might document the establishment of new professional networks (both formal and informal) of scientists and ethicists (such as the International Neuroethics Society), and the ways in which calls for regulation and ethical oversight can function as a means of rendering particular sociotechnical pathways legitimate.^31^ The legitimating function of bioethics has been analysed for ‘therapies’ rather than ‘enhancements’ (eg, using the case of pharmacogenetics), but any impact of (inter)national ethics bodies—if it exists—on the consumption of products that can be used to enhance bodily or cognitive function has not been formally examined.^32^ Further, we might also explore the degree to which the act of calling out issues of bioethical or regulatory concern can be employed as a vehicle for establishing organisational authority and legitimacy. In particular, under the Bush administration in the USA, the President's Council on Bioethics garnered considerable attention through bioethical agenda-setting and what some regard as overcautionary reports, including on enhancement.^33^ Both historical and ethnographic research could reveal how and to what extent ethical claims-making and organisational legitimacy relate to each other. Such scholarship could engage productively with feminist bioethics; this has cast a critical eye upon issues of authority and legitimacy, and renders problematic the universal subject that is often assumed by official ethics bodies. Rhetorical analyses of formal reports, documents and position papers deployed by such organisations will also illuminate embedded political rationalities (E Barr. The President's Council on Bioethics’ ‘Happy Souls’: discipline, citizenship and true selves, unpublished manuscript, 2014).

### Diverse bodies

Above we noted some of the diverse kinds of empirical attention that could be paid to the development, production and marketing of technologies and pharmaceuticals characterised in various spheres as enhancements. Cutting across this must also be an analytic sensitivity to the ways through which the intertwined issues of gender, ethnicity, sexuality, sociodemographic status and other markers of identity are implicated in practices of innovation and consumption.^34^ How are particular ideas of gender, for instance, embedded within enhancements, and how are new drugs and technologies gendered within media and marketing portrayals (and, indeed, within academic writing)? How do they impact on experienced and ascribed gender? Viagra is perhaps the case per excellence here, and empirical, STS-inflected research on this has cast bright light on its gendered marketing and uptake—but also on sites of resistance, and communities of users (eg, women) who have appropriated this drug and re-inscribed its embedded scripts so as to situate it within new regimes of personal life.^35^ Likewise, painkillers have been used in Indonesia to enhance libido, and hormonal therapies have been documented as being repurposed by individuals self-identifying as men who seek to feminise their bodies.^36^ Research in this vein reveals the complex social lives of ‘enhancements’ that exceed anticipatory imaginaries of their uses and actions, and that also enrich medical humanities and social scientific understandings of the relationships between biomedicine and wider society.^37^

A concern with gender—as well as ethnicity and other somatic markers of identity—connects with themes of embodiment more generally. Debates in this area might stimulate a consideration of any changes to how bodies ‘dys-appear’ (ie, come to subjective attention through pain, disability or impairment), disappear and reappear in societal practices and discourses associated with the drugs and technologies that some deem to enhance human traits and states.^38^ What aspects of ‘normal’ bodies could come to be regarded as sites of intervention in contexts where diverse forms of customisation are possible? The use of human growth hormone to simultaneously ‘treat’ and ‘enhance’ height is one example where the differences between ‘therapy’ and ‘enhancement’ emerge as complex, contingent and readily reworkable. With a focus on the ‘treatment’ of short boys, such distinctions are also highly gendered.^39^ Further, the human brain seems often to only become salient in personal discourse through its dys-appearance due to injury or cognitive impairment—yet, in an era of proliferating enhancements, it may increasingly be viewed as a plastic organ operable upon through techniques of ‘objective self-fashioning’ via pharmaceuticals or electroceuticals.^40–43^

At the same time, ‘dys-appearing’ forms of (especially) neurological difference are increasingly subject to projects of de-stigmatisation (eg, in the case of autism).^44^ In this light, understandings of techniques that operate upon human difference (be those drugs, devices or psychological interventions) can shift from being conceptualised as part of a therapy-enhancement continuum to being questioned over whether they are unwanted (and perhaps even coercive) tools of normalisation. The use of enhancements therefore represents the opening up of a new set of cases for empirical examination both in terms of how ‘differences’ between humans come to be recognised as such, and of how much difference populations are prepared to accept. Historians, anthropologists, geographers and others are particularly well placed to parse out how the uses of enhancement technologies might recast what bodily properties are taken as valued in particular contexts, by who and to what ends, and how the practices and consequences of valuation interact with actual engagements with enhancing technologies.^45^ A reinvigoration of research around cosmetic surgery is one starting point for the interrogation of these questions, especially including non-Western spaces and places where empirical ethnographic research might render problematic any assumptions that emerge regarding such practices based on their contemporary uses in Europe and North America.^46^ In Brazil, for example, the experimental use of testosterone to enhance vigour and verve means that there is no ready place to position this along any kind of linear ‘therapy’/‘enhancement’ continuum.^47^ Rather, as Alex Edmonds puts it, ‘health and aesthetics become entangled’.^48^ Cross-cultural empirical research could further illustrate (and perhaps contribute to the destabilisation of) Anglo-American societal norms that assume what counts as ‘therapy’ and what comprises ‘enhancement’ (simultaneously decentring ‘the West’ within bioethical analyses in this area).

### Constructing communities and contexts

Thinking about changing cross-cultural experiences and identities also directs our attention to the formation of new social groups orientated around apparent enhancements. Debates around the uses to which objects deemed enhancing might be put have helped to stimulate a range of neologisms (such as ‘bioconservative’ and ‘transhumanist’) that will be of interest to researchers keen to document, explore and understand how these become an idiom through which communities coalesce and expand. There are identity politics at play that are important to attend to: different communities can and could respond to discourses and practices centring on drugs and devices associated with human enhancement in diverse ways that speak to broader (and diverging) conceptions of humanity, sociality and public good.^49^ Disability studies scholars have begun to unpick some of the wider moral and political dimensions and implications of the imaginaries fuelling the speculation and advocacy of self-identifying ‘transhumanists’ (individuals who propose widespread adoption of a range of radical enhancements), for example. Their concerns regarding new forms of discrimination are suggestive of the need for empirical work with normative bite.^50^ Further, philosopher Trijsje-Marie Franssen has examined how the myth of Prometheus features within and configures debates around human enhancement and transhumanism (T-M Franssen. Prometheus through the ages: from ancient trickster to future human. University of Exeter: Unpublished doctoral thesis 2014. Available at: https://ore.exeter.ac.uk/repository/bitstream/handle/10871/15889/FranssenT.pdf?sequence=1). Scholarship of this kind provides insight into how images and imaginaries come to be instantiated within the identities of novel communities, and the discussions about medical technologies that these in turn propel.

A further point of departure for empirical work around the broad issue of ‘enhancement’ might be to interrogate how ethical debates and social practices (including but not limited to processes of innovation) mutually shape one another.^51^ The co-productionist tradition of STS—which foregrounds the dynamism between materiality and sociality—could provide rich conceptual resources for consideration of the reciprocal constitution of objects, moral discourse and social practices.^52^ Co-productionist studies of enhancement might also attend to how any routine use of enhancements could *change* their cultural contexts. How, for instance, does the promotion and use of particular technologies—such as drugs to enhance cognition—respond to issues of social concern (eg, educational attainment)? In turn: how, and to what extent, does the embedding of enhancements within societies recast the meanings of the terms by which societal issues are debated? Engagement with such a question necessarily entails an historical, rhetorical and ethnographic analysis of what ‘individual attainment’ is taken to be within specified cultures, and what cultural actors judge to be legitimate means of performing accomplishments. Accordingly, research that takes the practices of and politics around enhancement as a starting point could produce textured and nuanced scholarship that contributes to conceptual and empirical literatures far beyond the case study under primary examination.

Finally, a co-productionist perspective also enjoins us to engage with the emergence of the debate around ‘therapy’ and ‘enhancement’ itself. As indicated above, we argue that these concepts come to find meaning through particular configurations of social conventions, epistemic norms and biomedical tools. Intrinsically normative characterisations, their position in relation to each other is ambiguous and ever-changing, since the terrains of treatment and of enhancement have boundaries that shift according to mutations in the same sociotechnical practices that constitute them.^6^ ^10^ The flexibility of these spaces underscores what we regard as a central problem for medical humanities and social science scholars: how, why and where do particular technologies come to count as ‘enhancing’? Since the categories of ‘therapy’ and ‘enhancement’ are emergent through social praxis, any definitional settlements are of course essentially contingent, yet historical, geographical and anthropological research tracing processes of stabilisation is both timely and important.

## Empirical analyses of pharmaceutical cognitive enhancement

Throughout our analysis, we have sought to take care over how we use the term ‘enhancement’, indicating what empirical research agendas might be propelled through an acknowledgement that there are a number of individuals and groups who actively use this word and ascribe it to actual or imagined objects around which social practices are orientated—as well as many more people who (seek to) ‘enhance’ their bodies and brains in the absence of this idiom. In addition to being of import for the wider medical humanities and social sciences, we believe (and in so doing take cues from scholars such as Adam Hedgecoe) that empirical attention to the discourses and objects associated with enhancement might also better ground bioethical claims and appraisals in this area.^9^ Given that qualitative and quantitative methods have become more common in bioethics, we suggest that these methods can help to illuminate how ethically significant issues related to enhancement play out in everyday life.^53^ Practices of enhancing cognition, for example, have been central to much of the bioethical discussion that focuses explicitly on enhancement, especially the use of pharmaceuticals that promote concentration and alertness (for instance, brand names Adderall and Ritalin). Bioethicists of a more libertarian bent argue that such drugs should be more widely and freely available to those who want them, and that potentially citizens have a duty to consume them.^14^ Arguments like these can be seen as situated within a broader discourse of what social scientists have referred to as ‘biological citizenship’—the implicit or explicit connection of citizenship to somatic states or processes.^54^

Work is being produced that is seeking to quantify the degree to which drugs like Adderall are being used (eg, by university students).^55–57^ Interview studies are concerning themselves with the texture of this use and the meanings that are ascribed to it.^26^ ^58–61^ Notably, for instance, a recent paper by Singh *et al*^57^ underscored that many UK students were not familiar with cognitive enhancement. Such scholarship is needed in order to ensure that bioethical analysis is grounded in what seems to actually be happening in schools, workplaces and in the home—and what feasibly, based on empirical research on current practice, could start to happen.^7^ This is in contrast to a common bioethical focus on more speculative matters, and by commentators who may be geographically, professionally and personally distant from the figures that populate their imaginaries.

One useful resource is Catherine Coveney's sociological study of a range of individuals (including university students, health professionals and other shift workers) asked to envision the use of a drug promoting wakefulness (specifically, modafinil; brand name: Provigil).^6^ ^7^ Her findings showed that potential uses of this enhancement included ensuring safety at work (eg, ameliorating the dangers posed by sleepy surgeons), but also as a study aid. However, perceptions both of legitimate use and the legitimacy of the drug per se were tightly interwoven with its legality, and how readily available it was (eg, from local pharmacists, without a prescription, or from physicians only).^62^ Accordingly, what Coveney's research shows is that public attitudes towards enhancement drugs are linked to broader feelings towards pharmaceuticals more generally—and, specifically, the ways in which (to borrow historian Keith Wailoo's term) the ‘identities’ of drugs are constituted through regulatory regimes.^63^ ^64^ This insight has salience for bioethical debate that might take the meanings of pharmaceuticals to be solely a function of personal mores, as opposed to being powerfully shaped by the social frameworks in which they and their consumers are embedded.^65–67^

Other work by social psychologist Ilina Singh directs attention to the actual users of pharmaceuticals. One major theme around cognitive enhancement is ‘authenticity’, and bioethical scholarship focusing on this has until recently operated rather tangentially from empirical research. Questions discussed within the bioethics literature include what it might mean to live authentically, whether regular pharmaceutical use necessarily results in an ‘inauthentic’ life and if medication can in itself enhance authenticity. Conclusions may seek to direct the use of enhancements, including both restrictions and expansions of access to these drugs and technologies.^68^ A major contribution from Singh is her qualitative work with children diagnosed with ADHD, in which discussions took place around the relationship between psychopharmaceutical consumption and understandings of authenticity.^69^ ^70^ For this work, Singh interviewed more than 150 children between 9 and 15 in both the USA and UK. As she shows, drugs are not viewed as necessarily challenging or compromising characteristics associated in the bioethics literature with authenticity, and in some cases are figured by children as supporting, for instance, moral agency.^70^ This is suggestive of the degree to which the use of psychopharmaceuticals that act on cognition by consumers who do not have a psychiatric diagnosis may also refrain from seeing the use of Adderall or Ritalin as compromising their autonomy. There are implications here for bioethical debates around whether individuals should imbibe these substances in order to preserve their authenticity. Empirical work on authenticity potentially is of interest to philosophers concerned more directly with this concept per se.

Perspectives from users of ‘enhancements’ have also been the focus of sociologist Scott Vrecko's research.^26^ ^61^ His participants were university students at a US institution who used Adderall and Ritalin as study aids. Vrecko asked young men and women about their experiences of consuming such drugs, and elicited striking narratives of their practices. In particular, he revealed the diverse kinds of illicit exchanges through which prescription drugs come to be used for non-prescribed purposes, as well as the highly emotional impact of drug effects, whereby his respondents detailed how it felt to be ‘on’ psychopharmaceuticals that were understood by them to improve their work. Students discussed feelings of channelled interest and enjoyment (ie, a tight focus on, and pleasure in, the studies they were immediately employed in and not other things happening around them), ‘drivenness’, and feeling mentally and/or physically ‘up’.^61^ Such findings resonate with the more recent research of Margit Anne Petersen and colleagues, who likewise have underscored the role of prescription stimulants in sharpening students’ focus on their work, as well as on the subjective experience of work per se (and also on the moral ambivalences that characterise drug use in this context).^59^ ^60^ These experiences challenge the more ‘unemotional’ (as Vrecko puts it), sometimes strongly rationalist, accounts of decision-making and use that have found a home within much bioethical deliberation on cognitive enhancement. Hence, Vrecko's and Petersen *et al*'s findings are suggestive of the need to bring in the perspectives of actual users into the discursive spheres of bioethical and policy deliberation.

Work such as that summarised above thus complicates bioethical claims-making by underscoring the ways in which multiple meanings adhere to substances. As Coveney, Singh, Vrecko, Petersen and others remind us, drugs are never *just* their chemical constituents. Rather, they may take on a variety of ‘identities’ (and have different effects) depending on the context within which they are (or realistically might be) used. Many standard analyses of risk and benefits of pharmaceutical means of enhancing cognition take for granted what a drug ‘really’ is; that is, they are concerned solely with its active ingredients, mode of biological action and physiological effects. In so doing, commentaries and appraisals can elide the diverse and shifting societal understandings of the nature of the substance in question.^6^ ^8^ Besides holding science and society as separate domains, in these cases recommendations for policy and practice can be problematic as they might lack social salience. If a culturally impoverished understanding of a drug is mobilised in debate, the complexities of deciding whether or not this substance can, should or must be used are obfuscated, and so the legitimacy and utility of any conclusions reached are lessened. Our point is one that will be familiar to scholars concerned with the social lives of pharmaceuticals: that understandings of drugs and their effects are mediated through their use, the context of this, and who drugs are being used by and for what reasons. This illustrates the necessity of the empirical studies of enhancement already being undertaken. Further, it suggests the need for more in-depth quantitative, qualitative and interpretive research (informed by relevant theoretical and conceptual literatures from the medical humanities and social sciences) that can attend more precisely to the contextual issues we believe are so necessary to integrate into bioethical deliberation, and which have begun to be charted by the authors discussed above.^2^ ^3^ ^10^ ^29^ ^71^ ^72^

## Conclusion

Our aim in this analysis has been twofold. First, and most importantly, in common with scholars like Coveney, Morrison, Singh and Vrecko, we want to more firmly fix the attention of the empirical medical humanities and social sciences to the objects, bodies, subjectivities and discourses that populate debates around the enhancement of human bodies and minds. Our hope is that this can be achieved in ways that are not dismissive of traditional normative analysis or the debates around autonomy and responsibility that are so prevalent within bioethics, but which nevertheless uncover new territory for empirical exploration and theorisation. Indeed, perspectives from the wider medical humanities and social sciences can, we believe, also be brought to question the very terms through which bioethicists, policymakers, entrepreneurs and others are currently framing and advocating their positions and movements (eg, as enhancement ‘bioconservatives’ or proponents). Examining substances and devices that some regard as having the capacity to enhance human bodies and cognition can inform our understandings of the interactions between biomedicine and society more broadly, and hence, represent important case studies for scholars in cultural studies, history, law and STS, to name just a few disciplines.

Second, in mapping the terrain that the empirical medical humanities and social sciences might chart (and, indeed, have begun to explore), we want to make a renewed call for the importance of drawing the expertise of these scholars into the spheres of policy-orientated bioethical decision-making (and potentially disrupt them). We recognise the quality and rigour of many contributions from philosophers and others to understandings of enhancements in society; nevertheless, we suggest that a still more nuanced understanding of these issues, incorporating (or at least, drawing on) the expertise of other humanities scholars and of social scientists, might further enrich bioethical discussions. We believe that the appraisal and analysis of any new sociotechnical practices associated with enhancing human capabilities must be constituted through sensitivity to the larger historical, cultural and political context within which they find form—and likewise should be sensitive to how technologies of enhancement might reconfigure those contexts.^3^ ^6^ ^8^ ^10^ ^73^ Key here is research that engages a range of publics with decision-making around enhancements.^74^ ^75^ In so doing, there is an increased likelihood that recommendations can be reached which resonate with the lived experiences of policymakers, scientists, clinicians, patients and a range of other users. Accordingly, workable solutions to the governance of contested drugs and devices associated with enhancement might more readily be achieved.

## Footnotes

i We place the word ‘enhancement’ within quotes in order to signal our reticence to ontologise particular tools and substances as intrinsically ‘enhancing’ (or, indeed, ‘therapeutic’). Our reasons for this will become clearer as the analysis unfolds.

ii Specifically, light-responsive DNA segments are inserted into cells and when externally activated by a light source, can be switched on or off to observe effects in living subjects, or modulate cell function.
